# A descriptive analysis of the coverage of newborn care services among women who delivered in health facilities in 17 sub-Saharan African countries

**DOI:** 10.1186/s12884-023-05592-8

**Published:** 2023-04-17

**Authors:** Siyuan Wan, Baiming Jin, Mary Rachael Kpordoxah, Abdul-Nasir Issah, Daudi Yeboah, Jevaise Aballo, Michael Boah

**Affiliations:** 1 Department of Preventive Medicine, Qiqihar Medical University, Qiqihar, Heilongjiang China; 2 Department of Global and International Health, School of Public Health, University for Development Studies, Tamale, Ghana; 3 Department of Health Services, Planning, Management, and Economics, School of Public Health, University for Development Studies, Policy, Tamale, Ghana; 4 Department of Epidemiology, Biostatistics, and Disease Control, School of Public Health, University for Development Studies, Tamale, Ghana; 5 United Nations Children Fund (UNICEF), Ghana Country Office, P.O. Box AN 5051, Accra, Ghana

**Keywords:** Newborn care, Postpartum, Skin-to-skin contact, Breastfeeding, Sub-saharan Africa

## Abstract

**Background:**

Sub-Saharan Africa (SSA) has seen an increase in facility-based births over the years. However, the region has the world’s highest newborn mortality rate (42% in 2019). Quality care around the time of birth can avert these deaths. This study examined the newborn care interventions given to women who gave birth in health facilities in 17 countries in SSA.

**Methods:**

A cross-sectional population-based study was conducted. We used data from the most recent Demographic and Health Surveys (DHS) conducted in 17 sub-Saharan African countries. We analysed a weighted sample of 226,706 women aged 15–49 years who gave birth in the five years preceding the surveys. We described the coverage of nine newborn care services, namely weighing at birth, breastfeeding initiation within 1 h after birth, skin-to-skin contact, temperature measurement, cord examination, counselling on newborn danger signs, counselling on breastfeeding, breastfeeding observation, and child health assessment before discharge.

**Results:**

Overall, 72.0% (95% CI: 71.1, 72.8) of births occurred in health facilities, ranging from 40.0% (95% CI: 38.0, 42.1) in Nigeria to 96.3% (95% CI: 95.4, 97.1) in South Africa. Weighing at birth was the most common intervention (91.4%), followed by health checks before discharge (81%). The other interventions, including those given immediately at birth (breastfeeding and skin-to-skin contact), had suboptimal coverage. For instance, 66% of newborns were breastfed within 1 h after birth, and 56% had immediate skin-to-skin contact. Service coverage varied considerably by country and healthcare provider type.

**Conclusions:**

The majority of the examined services, namely early breastfeeding, skin-to-skin contact, cord examination, temperature measurement, counselling on newborn danger signs, breastfeeding observation, and counselling on breastfeeding, were found to have suboptimal coverage. Even though many pregnant women in SSA give birth in healthcare facilities, some newborns do not always get the care they need to be healthy and live. This is a missed chance to improve newborn health and survival around the time of birth.

**Supplementary Information:**

The online version contains supplementary material available at 10.1186/s12884-023-05592-8.

## Background

Newborn survival has improved. From 37 deaths per 1,000 live births in 1990 to 17 deaths per 1,000 live births in 2019, the global neonatal mortality rate went down by 52%, though it went down more slowly than deaths among children 1–59 months old [1]. In 2019, an estimated 2.4 million children died in their first month of life, with more than half of neonatal deaths occurring in the first 3 days of life and about two-thirds of these deaths occurring on the first day alone [1, 2]. Most of these deaths are preventable and occur in low- and middle-income countries (LMICs). Sub-Saharan Africa (SSA) alone accounted for 42% of all newborn deaths in 2019, the highest globally [1].

Preterm delivery complications, intrapartum events like birth asphyxia, or infections like sepsis or pneumonia account for almost 80% of newborn mortality [3].The Every Newborn Action Plan (ENAP) was adopted in June 2014 in response to the increasing focus on newborn mortality. The ENAP emphasised the strategic importance of focusing on quality care around the time of delivery. This includes ensuring that all pregnant women have access to the skilled quality care required for a healthy pregnancy, newborn protection, and care for small and sick babies [4]. This also aligns with the World Health Organization’s (WHO) recommendation on having a skilled attendant at birth to reduce mortality. However, the benefits of a skilled attendant at birth in reducing neonatal mortality are most clearly seen in the services provided during labour and delivery to prevent deaths and improve survival [5]. Therefore, concentrating on the period just before birth with crucial interventions that have been demonstrated to have a significant impact and providing good care for infants who are small or ill could prevent up to 80% of newborn deaths [3]. Essential newborn care includes care right after the baby is born and care throughout the first few weeks of life, such as delaying cord clamping, drying the baby well, checking the baby’s breathing, skin-to-skin contact, and breastfeeding as soon as possible [6, 7].

A significant portion of the extant scholarly works in Sub-Saharan Africa (SSA) have been dedicated to the investigation of antenatal care (ANC), comprising its scope of coverage, frequency of utilization, and the factors influencing its use [8–11]. Similarly, considerable attention has been paid to the locale of childbirth, particularly its extent of coverage, and the determinants of facility-based delivery [11–14]. We also found studies that examined services provided during the postpartum period, but their scope was limited. Benova and company, for instance, examined postnatal checks for women who gave birth in health facilities in SSA [15]. The focus of their study was whether the mother received a check-up by a health provider while in the facility and before discharge. Tessema and colleagues also investigated the determinants of postnatal care check up by health professionals within 42 days after birth among women in SSA [16]. As a result, there is still a lack of information about the coverage of essential services given to mothers and their newborns in health facilities after delivery to improve the health of newborns.

The current study used data from the most recent Demographic and Health Surveys (DHS) from 17 countries in SSA to examine the extent of coverage of certain newborn care interventions for women who gave birth at health facilities during the postpartum period. More specifically, the investigation focused on determining the percentage of postpartum women and their newborns who received essential services, including timely initiation of breastfeeding, skin-to-skin contact, examination of the umbilical cord, and measurement of body temperature, at the healthcare establishments where delivery occurred.

## Methods

### Study design, setting, and source of data

We used the most recent DHS, which was conducted in 17 SSA countries. These countries collected data on all the examined interventions, making them suitable for the present study. The DHS, funded by the United States Agency for International Development (USAID), collects information on a wide range of health indicators for the population, with a strong focus on the health of women and children. So far, the DHS is the primary source of information on the population-level coverage of health interventions for newborns. Standardized questionnaires are used in these surveys, ensuring that the information collected can be compared across countries. Strategies and methods for sampling have been explained previously [17].

### Participants

Our target population consisted of all women of reproductive age (15–49 years) who had given birth in the past 5 years preceding the surveys. After appending, the dataset contained 230,127 observations. However, we included 226,706 women who gave birth vaginally or via caesarean section; that is, 3421 observations were excluded because the place of delivery could not be reliably identified. The sample was weighted for the final analysis. The survey data ranged from 2011 to 2020.

### Interventions examined

In the DHS, women who gave birth were asked about the postpartum care they received while at the delivery facility. We incorporated nine interventions: (1) weighing of the newborn at birth; (2) immediate skin-to-skin contact; (3) timing of breastfeeding initiation; (4) examination of the newborn’s umbilical cord; (5) measurement of the newborn’s temperature; (6) counselling of the mother on danger signs for newborns; (7) counselling of the mother on breastfeeding; (8) observing the newborn breastfeeding; and (9) checking the baby’s health before discharge. We acknowledge that essential newborn care services encompass a broad range of services, including but not limited to delayed cord clamping, infection prevention, thorough drying, nurturing care, and timely and safe referral when needed, among others [6, 7]. However, we chose services that were measured in most (17 countries) of the countries in SSA.

### Statistical analysis

In the current study, descriptive statistics were used. In each country, the percentage of women who delivered their most recent baby in a health facility was calculated. Home deliveries, including those performed by traditional birth attendants (TBAs), are births that took place outside of healthcare institutions. Then we used a design-based Chi-square analysis on only the pooled data to compare the characteristics of women who delivered in healthcare facilities against those who delivered at home or with TBAs. We estimated the coverage of each newborn care intervention as the percentage of mothers who reported that the intervention was provided at the delivery healthcare facility. This analysis was restricted to only women who reported giving birth in healthcare facilities (N = 163, 185). All the analyses were carried out using STATA/IC 15.0 for Windows (StataCorp LLC, College Station, TX 77,845, USA). A probability value (*p*-value) of less than 0.05 was considered statistically significant. We used weighting factors to account for the DHS’s non-proportional sampling methods in its surveys [17]. In the analysis, the “svset” and “svy” commands were used to apply the complex survey design in our estimations.

### Ethical considerations

The current study analysed de-identified secondary data from the DHS. Therefore, no approval from an Institutional Review Board (IRB) was required. However, the DHS programme received ethical approval from both the Inner City Fund IRB and the IRB of the host nation. The surveys are conducted in accordance with the principles and ethics of health research involving human subjects. You can find the rules for protecting the privacy of survey respondents and household members in all DHS surveys 0n the DHS website (https://dhsprogram.com/Methodology/Protecting-the-Privacy-of-DHS-Survey-Respondents.cfm). We had access to the raw datasets used in the current study after obtaining written approval from the DHS program through the ICF.

## Results

### Prevalence of health facility delivery in the 17 sub-Saharan African countries

A weighted sample of 226,706 women aged 15–49 years who delivered vaginally or by caesarean section was analyzed. According to the results, the percentage of women who delivered in health facilities varied by country. Health facility delivery ranged from 40.0% (95% CI: 38.0, 42.1) in Nigeria to 96.3% (95% CI: 95.4, 97.1) in South Africa. The pooled results indicated that 72.0% (95% CI: 71.1, 72.8) of the deliveries occurred in health institutions. In six (35.3%) of the seventeen countries, that is Angola, Congo, Guinea, Mali, Nigeria, and Tanzania, health facility delivery was below the average of 72.0% (Table 1).

**Table 1:** Prevalence of health facility delivery in the 17 sub-Saharan African countries (N = 226,706)

Country	Year of survey	Total women analyzed	Health facility delivery % (95% CI)
Angola	2015-16	13,218	46.2(43.0, 49.4)
Benin	2017-18	13,446	85.3(82.9, 87.5)
Burundi	2016-17	13,075	87.6(86.3, 88.7)
Congo	2011-12	10,062	67.2(62.8, 71.3)
Gambia	2019-20	7,581	84.7(82.7, 86.4)
Guinea	2018	7,890	52.6(49.0, 56.2)
Liberia	2019-20	5,235	80.4(77.3, 83.2)
Malawi	2015-16	17,176	92.8(91.8, 93.7)
Mali	2018	10,286	67.1(63.0, 71.0)
Nigeria	2018	33,711	40.0(38.0, 42.1)
Rwanda	2019-20	8,217	94.6(93.6, 95.4)
Senegal	2019	5,600	80.8(76.6, 84.3)
Sierra Leone	2019	9,779	83.5(81.4, 85.6)
South Africa	2016	7,132	96.3(95.4, 97.1)
Tanzania	2015-16	19,802	63.7(60.5, 66.7)
Zambia	2018	19,506	84.8(82.8, 86.6)
Zimbabwe	2015	24,989	79.3(76.5, 81.8)
17 sub-Saharan African countries	2011–2020	226,706	72.0 (71.1, 72.8)

### Comparison of the characteristics of women who delivered in healthcare facilities in the 17 sub-Saharan African countries

Our pooled analysis revealed that women who delivered in health facilities in the 17 SSA countries differed significantly from those who delivered at home in terms of sociodemographic, economic, and obstetrical characteristics. Compared to their counterparts, a greater percentage of women aged 15–24 (73.3%) delivered in healthcare facilities compared to women in the age groups of 25–34 (72.6%) and 35–49 (69.1%). A higher percentage of women with at least secondary education (87.1%) and women who have never been in a union (84.3%) delivered in healthcare facilities compared to their counterparts. Regarding ANC use, 75.4% of women who used ANC at least once during their recent pregnancy delivered in healthcare facilities, compared to 17.8% of women who did not use ANC. A significantly higher percentage of women who resided in urban areas (84.0%) delivered in healthcare facilities compared to 65.6% of women in rural settings. A higher percentage of women from the richest households (93.4%) gave birth in healthcare facilities compared to women from households in the other wealth groups (Table 2).

**Table 2:** Comparison of the characteristics of women who delivered in healthcare facilities in the 17 sub-Saharan African countries (N = 226,706)

Variable	Frequency (weighted)	Place of delivery		*P*-value
		Home (%)	Health facility (%)	
Age group (Years)				< 0.001
15–24	64,915	26.7	73.3	
25–34	108,664	27.4	72.6	
35–49	53,127	30.9	69.1	
Highest educational level				< 0.001
No formal education	74,949	46.1	53.9	
Primary	76,607	25.2	74.8	
At least secondary	75,151	12.9	87.1	
Current marital status				< 0.001
Never in a union	16,577	15.7	84.3	
Married	164,453	29.3	70.7	
Living with partner	29,868	29.8	70.2	
Widowed	2,975	29.7	70.3	
Divorced	5,245	21.9	78.1	
Separated	7,589	23.9	76.1	
Number of children ever born				< 0.001
1–2	82,789	18.6	81.4	
3–4	72,353	27.3	72.7	
5 or more	71,066	39.7	60.3	
Number of living children				< 0.001
0	82,971	29.7	70.3	
1–2	72,512	20.2	79.8	
3–4	71,223	28.7	71.3	
5 or more	82,971	39.5	60.5	
Antenatal care use during recent pregnancy				< 0.001
No	13,353	82.2	17.8	
Yes	213,353	24.6	75.4	
Place of residence				< 0.001
Urban	78,100	16.0	84.0	
Rural	148,606	34.4	65.6	
Wealth quintile				< 0.001
Poorest	52,126	45.8	54.2	
Poorer	49,252	37.1	62.9	
Middle	44,832	27.1	72.9	
Richer	43,567	15.7	84.3	
Richest	36,930	6.6	93.4	

Coverage of essential newborn care services among women who delivered in health facilities in the 17 sub-Saharan African countries.

**Fig. 1:**
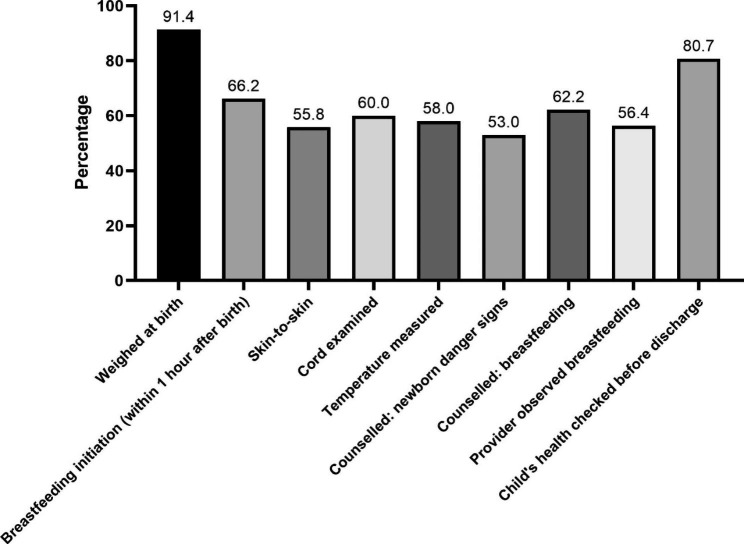
Coverage of newborn care services among women who delivered in health facilities in 17 sub-Saharan African countries (N = 163,185)

There were disparities in the coverage of newborn care services among women who gave birth in health facilities in the 17 SSA countries. Among the examined services, weighing at birth was commonly reported by 91.4% of the sample (ranging from 60% in Liberia to 99.1% in Rwanda and Zimbabwe), followed by checking a child’s health before discharge (80.7%). Counselling on newborn danger signs had the lowest coverage of 53.0% (ranging from 31.5% in Mali to 82.4% in Sierra Leone). The coverage rates for breastfeeding initiation within 1 h of birth and immediate skin-to-skin contact were 66.2% (ranging from 37.6% in Gambia to 86.8% in Rwanda) and 55.8% (ranging from 12.7% in Burundi to 86.7% in Benin), respectively (Fig. 1 and Additional file 1: Table S1). When the analysis excluded women who delivered through caesarean section (see Additional file 1: Table S1), we observed that early breastfeeding initiation was 68.8% (ranging from 38.8% in Gambia to 91.6% in Rwanda), while immediate skin-to-skin was 57.5% (ranging from 13.1% in Burundi to 89.1% in Benin).

### Coverage of essential newborn care services by healthcare provider type

We also analysed newborn service coverage by type of health care provider. According to the data (Table 3), the coverage of the following services was comparable between public and private health facilities: weight taking at birth, counselling on newborn danger signs, counselling on breastfeeding, and observation of breastfeeding. The coverage of services, such as breastfeeding initiation within 1 h after birth, skin-to-skin contact, cord examination, and temperature measurement, varied considerably between public and private health institutions. A greater percentage of newborns in private facilities than in public facilities have their cords examined, their temperatures taken, and their health checked before discharge. In private facilities, the rate of cord examination is 62.7%, compared to 59.6% in public facilities. Similarly, private health facilities measure newborn temperatures at a rate of 62.1%, compared to 57.4% in public health facilities. Also, the percentage of children’s health checked before discharge at private health facilities was 83.5% compared to 80.2% in public health facilities. In contrast, public health facilities had higher rates of early breastfeeding initiation within 1 h after birth and skin-to-skin contact coverage than private ones. For instance, initiation of breastfeeding within 1 h of birth was more prevalent (67.0%) in public health facilities than in private health facilities (60.97%). Similarly, the rate of immediate skin-to-skin contact was 56.5% in public health facilities and 51.1% in private health facilities (Table 3).

**Table 3:** Coverage of essential newborn care services by healthcare provider type (N = 161,862)

Service	Health provider type		*P*-value
	Public (n = 139,348) % (95% CI)	Private/mission (n = 22,514) % (95% CI)	
Newborn weighed at birth	91.5(91.0, 91.9)	90.8(89.9, 91.6)	0.095
Breastfeeding initiation (within 1 h after birth)	67.0(66.3, 67.7)	60.9(59.5, 62.2)	< 0.001
Skin-to-skin contact	56.5(55.7, 57.3)	51.1(49.4, 52.7)	< 0.001
Cord examined	59.6(58.7, 60.4)	62.7(61.0, 64.4)	0.001
Temperature measured	57.4(56.5, 58.2)	62.1(60.3, 63.9)	< 0.001
Provider counselled on newborn danger signs	52.9(52.1, 53.8)	53.7(52.0, 55.3)	0.384
Provider counselled on breastfeeding	62.1(61.2, 63.0)	62.7(61.1, 64.4)	0.437
Provider observed breastfeeding	56.3(55.4, 57.2)	56.9(55.3, 58.5)	0.458
Child’s health checked before discharge	80.2(79.7, 80.8)	83.5(82.3, 84.6)	< 0.001

## Discussion

The purpose of this descriptive study was to examine the coverage of nine newborn care interventions among women who delivered in healthcare facilities in seventeen countries in SSA. We discovered that more than two-thirds of deliveries took place in healthcare facilities, with Nigeria having the lowest rate and South Africa having the highest. The documented rate of health facility delivery in the current study is greater than the 66% reported by Adde et al. in their analysis of 28 countries in SSA [12]. The estimate of health facility delivery is based on a pooled analysis and may be influenced by country-level coverages. As a result, we suspect that the lower estimate reported by Adde et al.’s study, which rather included a relatively higher number of countries, was influenced by low prevalence rates observed in more than half of the included countries, which probably obscured the high coverages in the remaining few countries. However, similar to the current study’s findings, there were substantial variations in prevalence rates between countries. Based on their findings, the percentage of births that occurred in health facilities ranged from 23% in Chad to 94% in Gabon.

In the first 3 days after birth, more than half of neonatal deaths occur [2]. Reducing mortality during this period would require evidenced-based interventions such as postnatal care and care for small babies and sick newborns. Immediate care at birth, specifically early breastfeeding initiation within 1 h after birth and immediate skin-to-skin contact, improves newborn health and survival. Early breastfeeding initiation reduces the risk of neonatal morbidity and mortality [18–20]. Immediate skin-to-skin contact reduces neonatal hypothermia and maintains optimal body temperature [21]. Skin-to-skin newborns were more likely to be breastfed early than those who were not [21–23]. Feldman et al. also found that children who were put in skin-to-skin contact with their mothers had better autonomic functioning and cognitive control in their first 10 years of life [24]. The act of weighing neonates at birth could aid in the identification of infants with distinct healthcare requirements, notably those who are born with a low birthweight, warranting specific attention and interventions to improve their chances of survival. According to survey data from 20 SSA countries, babies weighed at birth are less likely to die within the first 27 days of life [20]. Despite these obvious benefits, the current study revealed that coverage of most essential newborn interventions, including those classified under “immediate care at birth” (initiation of breastfeeding within 1 h and skin-to-skin contact) by the WHO, is less than optimal across countries. Only weight taking at birth and assessing the child’s health at discharge had higher coverage rates among the interventions. It is important to note, however, that the current study’s coverage of early breastfeeding initiation and immediate skin-to-skin contact is higher than previously reported [25, 26]. We also found that coverage of five interventions: early breastfeeding initiation within 1 h after birth, immediate skin-to-skin contact, cord examination, temperature taking, and checking the child’s health before discharge differed substantially by provider type. Private facilities outperformed public health in providing quality primary health care according to a previous study conducted in the current study’s setting [27]. In the current study, private facilities had higher coverage in three of the five interventions that differed by provider type, thus outperforming public ones. However, newborns were more likely to receive early breastfeeding and skin-to-skin contact in public health facilities than in private facilities. This finding confirms earlier research indicating that babies in Uganda were more likely to receive thermal care practises in public facilities than in private ones [28].

The current study’s design prevented us from investigating the factors underlying the observed coverages. Furthermore, we believe that because women gave birth in healthcare facilities, any differences in services received may be related to the healthcare system in question. However, aside from the type of provider, the datasets used for the current analysis did not contain information about the healthcare system that we could investigate further. Nonetheless, existing literature suggests that a variety of factors, including service readiness, may influence the variation in service coverage between countries. An assessment of the health service environment revealed that newborns in areas with high service readiness are more likely to receive essential newborn care [29]. Major deficiencies in essential newborn care supplies and equipment, health care worker density, as well as in health worker knowledge and performance of key routine newborn care practices, especially for immediate skin-to-skin contact and breastfeeding initiation, were also identified in a cross-section of health facilities across six eastern SSA nations: Ethiopia, Kenya, Madagascar, Mozambique, Rwanda, and Tanzania [30].

The facility’s delivery of newborn services can be impacted by several factors, including the mode of delivery and other newborn outcomes, such as birth weight and Apgar score. Notably, cesarean deliveries were found to exhibit a decreased likelihood of early breastfeeding within the first hour after birth and immediate skin-to-skin contact, in comparison to spontaneous vaginal deliveries [22, 23, 31, 32]. Similarly, it was observed that women exhibited decreased likelihood to commence skin-to-skin contact and initiate early breastfeeding when their neonates presented with an Apgar score of less than seven at one minute post-delivery [33]. Low birthweight infants, on the other hand, were more likely to engage in immediate skin-to-skin contact with their mothers than babies born with a normal birthweight [25]. Furthermore, it was observed that singleton births were associated with a higher likelihood of initiating early breastfeeding compared to multiple births or twin deliveries [34]. As shown in the current study, coverages of early breastfeeding and immediate skin-to-skin contact increased when caesarean births were excluded in the analyses. Unfortunately, our analysis was unable to assess the potential influence of Apgar score at birth and birth type on these interventions.

Heterogeneity between the countries also explains the observed variations in the coverage of newborn services. In assessing the quality of primary health care services in seven countries across eastern, western, and southern Africa, Kruk and her colleagues found that differences between countries were more important than all other factors in explaining differences in care quality [27]. The quality of maternal and newborn care services in SSA differs by country due to a range of factors, including variations in health system infrastructure, health workforce availability and capacity, financing mechanisms, and political will. In countries where there is insufficient or weak health infrastructure, inadequate health workforce, limited health expenditure, and weak political commitment, the quality of healthcare services is often suboptimal [35].

### Interpretation

This study’s findings have implications for stakeholders and policymakers who are involved in neonatal and child health. First, the findings confirm that there is a gap in the quality of care during the critical period of a newborn’s life in some SSA countries. In particular, even though a substantial proportion of pregnant women in nearly half of countries in SSA utilize the health system to deliver, the essential interventions required to promote optimal health and survival of the newborn after birth are not always available, and even when they are, they are not always provided.

Second, there is an urgent need to increase the proportion of newborns receiving essential newborn care interventions in healthcare facilities during the critical period of life. SSA is particularly important given the high mortality across all age groups and the continuous population growth. The region is estimated to realise 446 million births by 2030 [36]. The rapid increase in the number of births and population necessitates increased investment in newborn and child survival interventions as well as strengthening the health systems that provide them, otherwise neonatal mortality in the region may stagnate or worsen. There are a variety of strategies to improve the coverage of newborn care interventions at the facility level in LMICs, including training for key staff, implementation of checklists or job aids, and task shifting [7]. Nonetheless, to be effective, these interventions need to be well-described and accompanied by systems for monitoring and reporting implementation outcomes.

### Study strengths and limitations

This study used nationally representative data from 17 SSA countries to investigate the coverage of essential newborn care services provided to women who deliver at health facilities during the postpartum period. This study adds to the literature on the quality gap during the postpartum period in these countries. However, although the current study provides useful information that could potentially, aid in the improvement of newborn care services in LMICs, some weaknesses have to be acknowledged. The current study has a descriptive design. As a result, we could only provide information on the coverage of the interventions examined; we were unable to examine the specific reasons for these coverages through analysis. Also, there are several newborn care services available. However, we only examined nine interventions because information on them was available for only 17 countries. It is imperative to acknowledge that these interventions must not supersede other measures such as infection control and neonatal resuscitation. It is unfortunate that the surveys conducted by DHS do not capture data on these interventions. We used secondary data from surveys of women who had their most recent baby within the previous five years. Women’s deliveries could have occurred up to three or four years before the survey, and they may not remember whether or not a certain service was provided. As a result, the possibility of recall errors cannot be ruled out. However, a study demonstrated that women could accurately recall maternal and newborn interventions received in the postnatal period [37]. Furthermore, we encountered a challenge in discerning between preterm and asphyxiated infants from healthy newborns using the dataset. Another limitation is that the DHSs are conducted on an ongoing basis, and the data from the participating countries in the current study were not collected at the same time, limiting contemporaneous cross-country comparisons by time. Nonetheless, we used data from the most recent surveys, which allowed us to learn about the most recent trends within these countries.

## Conclusion

The study found that, among the majority of the services examined, namely early breastfeeding, skin-to-skin contact, cord examination, temperature measurement, counselling on newborn danger signs, breastfeeding observation, and counselling on breastfeeding, coverage was found to be less than optimal. From the study, although a significant percentage of pregnant women in SSA give birth in health facilities, the essential interventions that are needed to promote newborn health and survival are sometimes not provided. The findings demonstrate a missed opportunity around the time of birth to promote newborn survival in many SSA countries.

## Electronic supplementary material

Below is the link to the electronic supplementary material.

**Additional file 1:** Table S1

## Data Availability

The data underlining the conclusions drawn in this study are contained within the manuscript. The dataset, however, can be obtained freely from the DHS website (https://dhsprogram.com/data/dataset_admin/index.cfm) with permission from the DHS program. The authors do not have the right to share the dataset with other researchers without approval from the DHS program.

## Abbreviations

ANC: Antenatal care
AOR: Adjusted odds ratio
CI: Confidence interval
DHS: Demographic and Health Survey
ENAP: Every Newborn Action Plan
IRB: Institutional Review Board
LMICs: low-and-middle income countries
SSA: sub-Saharan Africa
TBAs: Traditional Birth Attendants
USAID: United State Agency for International Development
WHO: World Health Organization
