# Supplementary material for: A descriptive analysis of the coverage of newborn care services among women who delivered in health facilities in 17 sub-Saharan African countries
Source: BMC Pregnancy Childbirth. 2023 Apr 17;23:256. doi: 10.1186/s12884-023-05592-8 (PMC10108479; doi:10.1186/s12884-023-05592-8)
Supplement: Supplementary file 1 — Additional file 1: Table S1 [file 12884_2023_5592_MOESM1_ESM.pdf]

Country-specific data on the coverage of newborn care interventions among women who delivered in healthcare facilities in 17 sub-Saharan African countries

Table 1: Country-specific data on the coverage of newborn care interventions among women who delivered in healthcare facilities in 17 sub-Saharan African countries

Country	Weighed at birth	Breastfeeding initiation (within 1 hour after birth)	Breastfeeding initiation (within 1 hour after birth): excluding caesarean births	Immediate skin-to-skin contact	Immediate skin-to-skin contact: excluding caesarean births
Angola	93.2(91.8, 94.5)	53.1(49.8, 56.3)	55.2(51.9, 58.5)	66.0(62.2, 69.5)	68.4(64.4, 72.0)
Benin	86.8(85.0, 88.5)	54.5(52.4, 56.6)	56.7(54.5, 58.9)	86.7(85.3, 88.0)	89.1(87.7, 90.4)
Burundi	91.2(90.0, 92.3)	85.6(84.6, 86.7)	89.2(88.1, 90.1)	12.7(11.5, 14.1)	13.1(11.8, 14.5)
Congo	95.6(94.7, 96.3)	55.3(52.3, 58.3)	56.7(53.8, 59.9)	57.4(54.5, 60.3)	59.3(56.3, 62.2)
Gambia	97.9(97.0, 98.4)	37.6(34.2, 41.2)	38.8(35.3, 42.4)	34.7(32.1, 37.3)	35.1(32.5, 37.8)
Guinea	85.7(82.8, 88.1)	53.2(49.8, 56.5)	54.9(51.5, 58.4)	36.5(33.3, 39.7)	36.5(33.2, 39.9)
Liberia	60.0(56.3, 63.7)	66.6(62.7, 70.3)	69.4(65.3, 73.2)	57.1(53.8, 60.3)	59.5(56.2, 62.8)
Malawi	96.3(95.8, 96.8)	78.9(77.6, 80.3)	80.8(79.4, 82.0)	68.7(67.2, 70.2)	70.1(68.5, 71.6)
Mali	70.4(66.7, 73.8)	69.5(67.0, 71.8)	71.2(68.8, 73.6)	31.3(28.3, 34.5)	31.7(28.6, 34.9)
Nigeria	63.8(61.5, 66.0)	51.9(50.2, 53.7)	54.6(52.9, 56.3)	20.1(18.5, 21.8)	20.9(19.2, 22.7)
Rwanda	99.1(98.9, 99.3)	86.8(85.6, 88.0)	91.6(90.6, 92.5)	80.2(78.9, 81.4)	88.2(87.1, 89.1)
Senegal	95.4(94.0, 96.5)	37.7(33.9, 41.7)	40.9(36.8, 45.0)	71.4(68.6, 74.2)	75.4(72.5, 78.1)
Sierra Leone	91.4(90.2, 92.5)	75.9(73.6, 78.7)	77.2(74.8, 79.33)	68.5(66.2, 74.2)	68.6(66.3, 70.8)
South Africa	98.9(98.3, 99.3)	77.1(74.5, 79.5)	79.8(76.8, 82.4)	69.1(66.3, 71.8)	72.2(69.1, 75.1)
Tanzania	95.8(94.8, 96.6)	58.0(55.8, 60.1)	62.8(60.6, 65.1)	45.0(42.7, 47.4)	47.5(44.9, 49.9)
Zambia	97.2(96.4, 97.8)	79.3(77.3, 81.2)	83.1(81.4, 84.7)	65.4(63.0, 67.7)	67.8(65.1, 70.5)
Zimbabwe	99.1(98.7, 99.4)	61.7(59.6, 63.8)	64.7(62.5, 66.7)	59.9(57.6, 62.2)	61.8(59.4, 64.1)
17 Sub-Saharan Africa	91.4(91.0, 91.8)	66.2(65.5, 66.8)	68.8(68.2, 69.5)	55.8(55.0, 56.6)	57.5(56.7, 58.3)

Table 1 continued.

Country	Cord examined	Temperature measured	Provider counselled on newborn danger signs	Provider counselled on breastfeeding	Provider observed breastfeeding	Child's health checked before discharge
Angola	51.1(47.8, 54.5)	49.6(46.2, 52.9)	48.9(45.6, 52.3)	57.3(53.7, 60.8)	47.1(43.4, 50.8)	62.9(59.6, 66.2)
Benin	51.3(49.0, 53.5)	55.5(53.4, 57.6)	42.9(40.9, 45.1)	51.1(48.9, 53.3)	43.7(41.5, 45.9)	83.1(81.3, 84.7)
Burundi	6.6(5.8, 7.6)	5.5(4.8, 6.2)	4.3(3.7, 4.9)	6.3(5.6, 7.1)	5.4(4.8, 6.2)	55.2(53.2, 57.1)
Congo	65.6(62.8, 68.3)	70.7(67.8, 73.4)	55.9(53.5, 58.5)	63.6(60.8, 66.4)	55.5(52.7, 58.3)	87.1(85.1, 88.8)
Gambia	69.4(64.9, 73.6)	68.4(64.0, 72.6)	49.4(46.1, 52.7)	50.0(46.5, 53.6)	43.7(40.5, 46.9)	96..1(95.2, 96.9)
Guinea	49.6(46.3, 52.9)	48.6(45.2, 52.0)	51.7(48.5, 54.9)	52.7(49.6, 55.9)	39.6(36.7, 42.5)	81.1(78.5, 83.4)
Liberia	55.9(51.9, 59.8)	51.3(46.9, 55.6)	53.3(49.4, 34.8)	58.8(54.8, 62.7)	54.1(50.2, 57.9)	90.0(88.4, 91.5)
Malawi	63.9(62.3, 65.5)	68.9(67.2, 70.5)	72.2(70.9, 73.5)	86.8(85.7, 87.8)	72.8(71.4, 74.2)	67.8(66.0, 69.4)
Mali	33.3(29.9, 36.9)	31.4(28.2, 34.8)	31.5(28.4, 34.8)	40.0(36.7, 43.4)	35.5(32.8, 38.3)	75.8(72.7, 78.6)
Nigeria	40.7(38.8, 42.7)	42.6(40.6, 44.6)	38.7(36.8, 40.5)	41.8(40.0, 43.6)	35.9(34.2, 37.7)	83.5(82.2, 84.7)
Rwanda	67.5(65.6, 69.4)	53.2(51.2, 55.1)	48.7(46.7, 50.7)	73.8(72.3, 75.3)	82.1(80.6, 83.4)	79.2(77.5, 80.9)
Senegal	75.0(71.3, 78.3)	74.2(70.4, 77.5)	50.8(47.6, 54.0)	56.9(53.5, 60.2)	44.5(40.1, 49.0)	99.7(99.5, 99.8)
Sierra Leone	84.1(81.9, 86.0)	83.8(81.6, 85.7)	82.4(80.2, 84.4)	84.2(82.2, 86.1)	76.2(73.7, 78.4)	93.0(91.4, 94.4)
South Africa	89.2(86.4, 91.4)	89.5(86.8, 91.7)	78.5(75.8, 80.9)	84.7(82.0, 87.0)	78.9(76.2, 81.5)	97.0(96.1, 97.7)
Tanzania	51.6(49.5, 53.6)	35.0(32.7, 37.3)	32.5(30.3, 34.9)	50.2(47.8, 52.7)	47.9(45.6, 50.3)	63.2(61.2, 65.1)
Zambia	64.5(62.0, 66.8)	61.0(58.4, 63.6)	58.4(55.8, 60.9)	64.3(61.8, 66.7)	60.4(57.8, 63.0)	84.1(82.5, 85.5)
Zimbabwe	81.7(79.7, 83.6)	79.2(76.7, 81.4)	70.9(68.7, 73.2)	84.9(83.1, 86.7)	80.4(78.4, 82.3)	91.3(89.9, 92.4)
**17 Sub-Saharan Africa**	**60.0(59.2, 60.8)**	**58.0(57.2, 58.9)**	**53.0(52.2, 53.8)**	**62.2(61.3, 63.0)**	**56.4(55.6, 57.2)**	**80.7(80.1, 81.2)**
